# Supplementary material for: HIV-1 control in vivo is related to the number but not the fraction of infected cells with viral unspliced RNA
Source: bioRxiv. 2024 Jul 2:2024.07.01.601579. Preprint. [Version 1] doi: 10.1101/2024.07.01.601579 (PMC12478367; doi:10.1101/2024.07.01.601579)
Supplement: Supplement 1 [file NIHPP2024.07.01.601579v1-supplement-1.pdf]

bioRxiv preprint doi: https://doi.org/10.1101/2024.07.01.601579; this version posted July 2, 2024. The copyright holder for this preprint (which was not certified by peer review) is the author/funder. This article is a US Government work. It is not subject to copyright under 17 USC 105 and is also made available for use under a CC0 license.

Supporting Information for

HIV-1 control in vivo is related to the number but not the fraction of infected cells with viral unspliced RNA

Adam A. Capoferri[1,2], Ann Wiegand[1], Feiyu Hong[3], Jana L. Jacobs[3], Jonathan Spindler[1], Andrew Musick[4], Michael J. Bale[1,5], Wei Shao[4], Michele D. Sobolewski[3], Anthony R. Cillo[6], Brian T. Luke[4], Christine M. Fennessey[7], Robert J. Gorelick[7], Rebecca Hoh[8], Elias K. Halvas[3], Steven G. Deeks[8], John M. Coffin[9,*], John W. Mellors[3], and Mary F. Kearney[1,*]

[1]HIV Dynamics and Replication Program, National Cancer Institute, Frederick, MD, USA
[2]Department of Microbiology and Immunology, Georgetown University, Washington D.C., USA
[3]Division of Infectious Diseases, University of Pittsburgh School of Medicine, Pittsburgh, PA, USA
[4]Leidos Biomedical Research, Inc, Frederick National Laboratories for Cancer Research, Frederick, MD, USA
[5]Laboratory of Epigenetics and Immunity, Department of Pathology and Laboratory Medicine, Weill Cornell Medicine, New York, NY, USA
[6]Department of Immunology, University of Pittsburgh School of Medicine, Pittsburgh, PA, USA
[7]AIDS and Cancer Virus Program, Frederick National Laboratory for Cancer Research, Frederick, MD, USA
[8]University of California San Francisco, San Francisco, CA, USA
[9]Department of Molecular Biology and Microbiology, Tufts University, Boston, MA, USA

Corresponding:
[*]John M. Coffin
Email: john.coffin@tufts.edu

[*]Mary F. Kearney
Email: kearneym@mail.nih.gov

bioRxiv preprint doi: https://doi.org/10.1101/2024.07.01.601579; this version posted July 2, 2024. The copyright holder for this preprint (which was not certified by peer review) is the author/funder. This article is a US Government work. It is not subject to copyright under 17 USC 105 and is also made available for use under a CC0 license.

Supplemental Figure 1

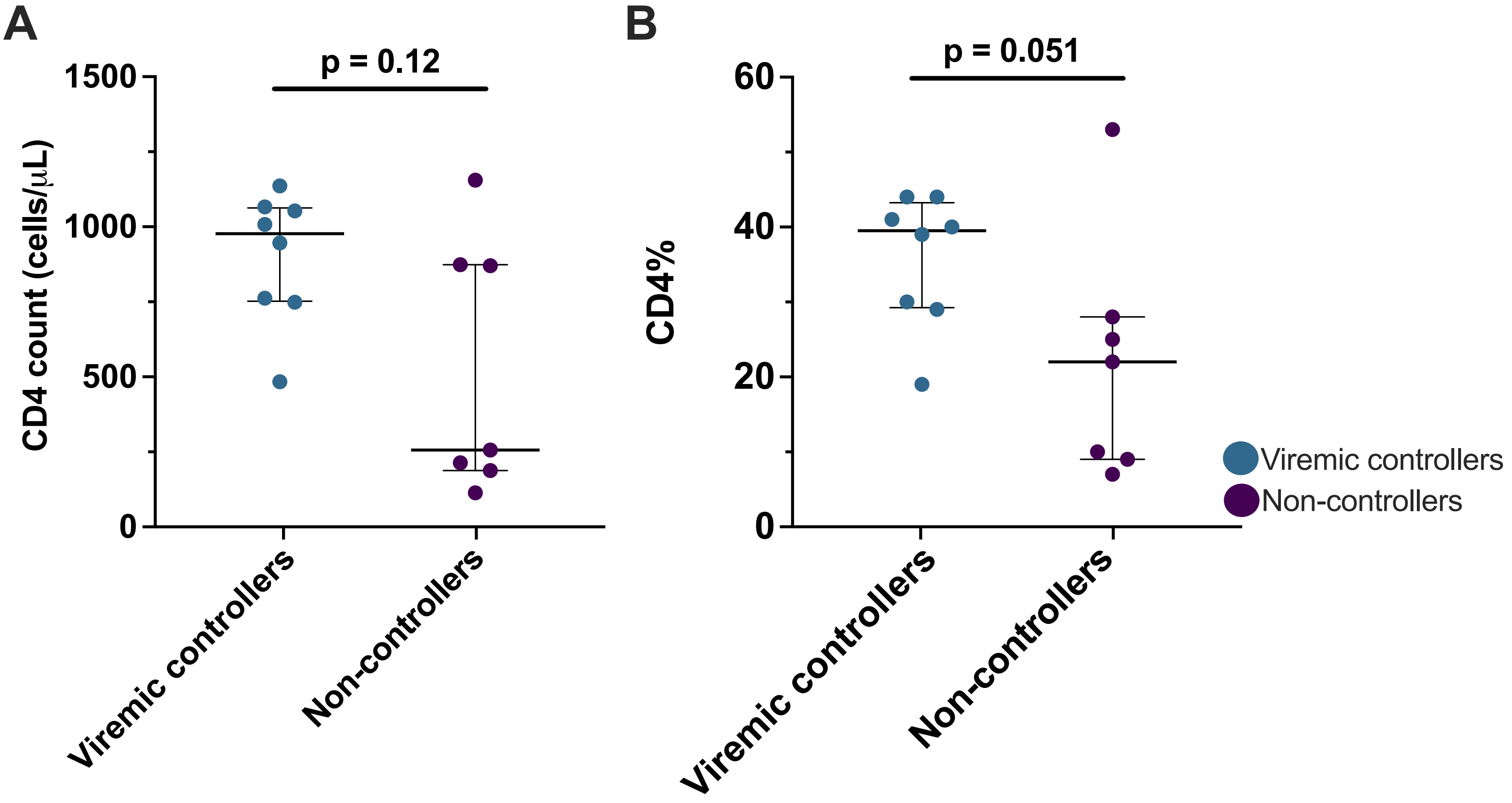

Supplemental Figure 1. *Measurement of CD4+ T cells in untreated donors.* Clinical assays using flow cytometry measured the (**A**) CD4+ T cell count as cells/μL, and (**B**) %CD4+ T cells in PBMC in whole blood as measured by flow cytometry. Mann-Whitney test was performed with the median and interquartile range reported. Each symbol represents an individual donor within the respective group. Measurements are at the time of sampling for **Table 2**.

bioRxiv preprint doi: https://doi.org/10.1101/2024.07.01.601579; this version posted July 2, 2024. The copyright holder for this preprint (which was not certified by peer review) is the author/funder. This article is a US Government work. It is not subject to copyright under 17 USC 105 and is also made available for use under a CC0 license.

Supplemental Figure 2

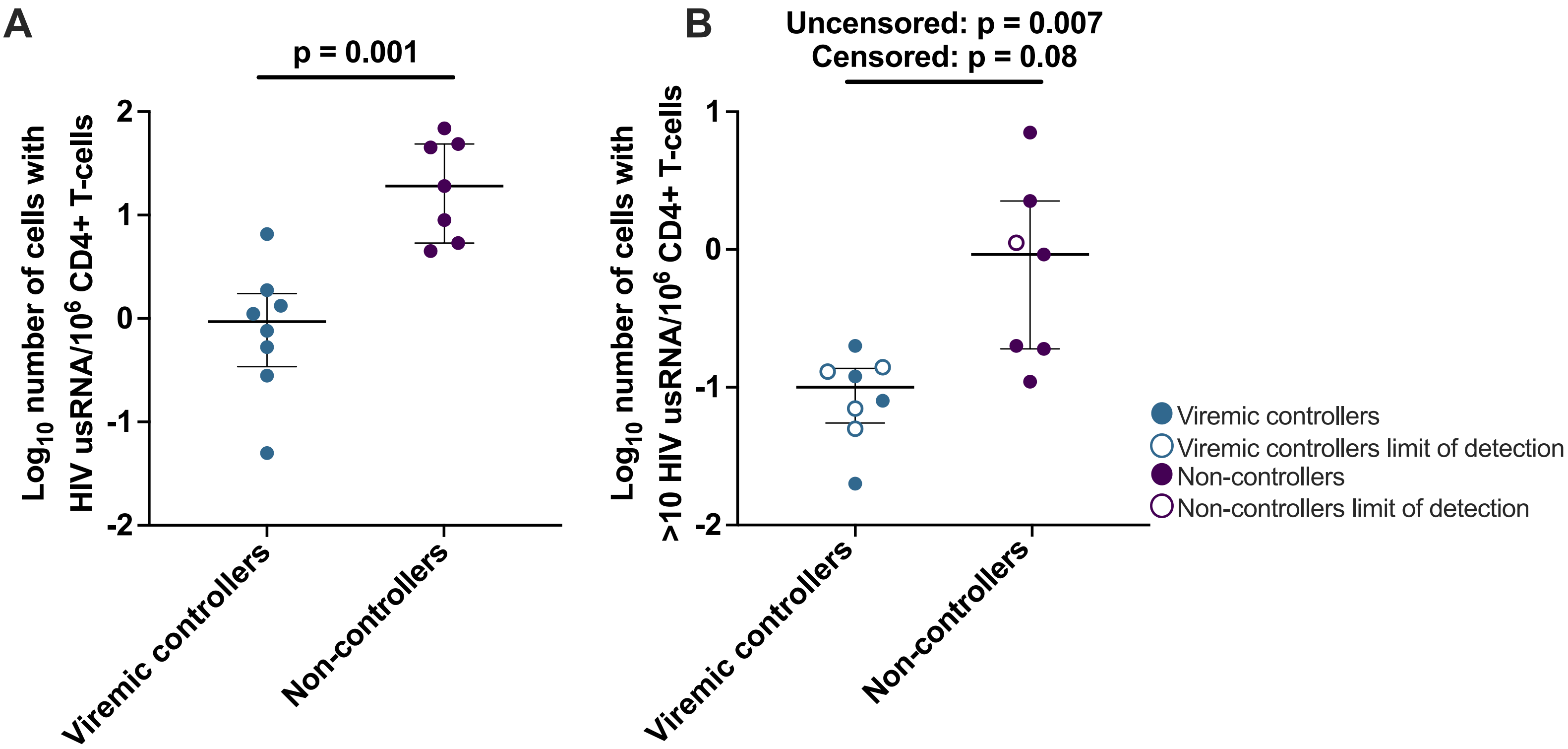

Supplemental Figure 2. *Number of cells with HIV usRNA normalized to CD4+ T cells.* **(A)** The number of cells with HIV usRNA per million PBMC was normalized to CD4+ T cells by the %CD4 at the time of sampling. **(B)** The number of cells per million PBMC that are "high-expressing" (>10 copies HIV usRNA) normalized to CD4+ T cells by the %CD4 at the time of sampling. Open shapes indicate no detectable "high-expressing" cells in a particular donor, plotted at the estimated limit of detection estimate determined as 1/number of PBMC assayed. Mann-Whitney test for both uncensored and censored data (donors with undetectable "high-expressing" cells, open shapes) with median and interquartile range as shown. Each symbol represents an individual donor within the respective group.

bioRxiv preprint doi: https://doi.org/10.1101/2024.07.01.601579; this version posted July 2, 2024. The copyright holder for this preprint (which was not certified by peer review) is the author/funder. This article is a US Government work. It is not subject to copyright under 17 USC 105 and is also made available for use under a CC0 license.

Supplemental Figure 3

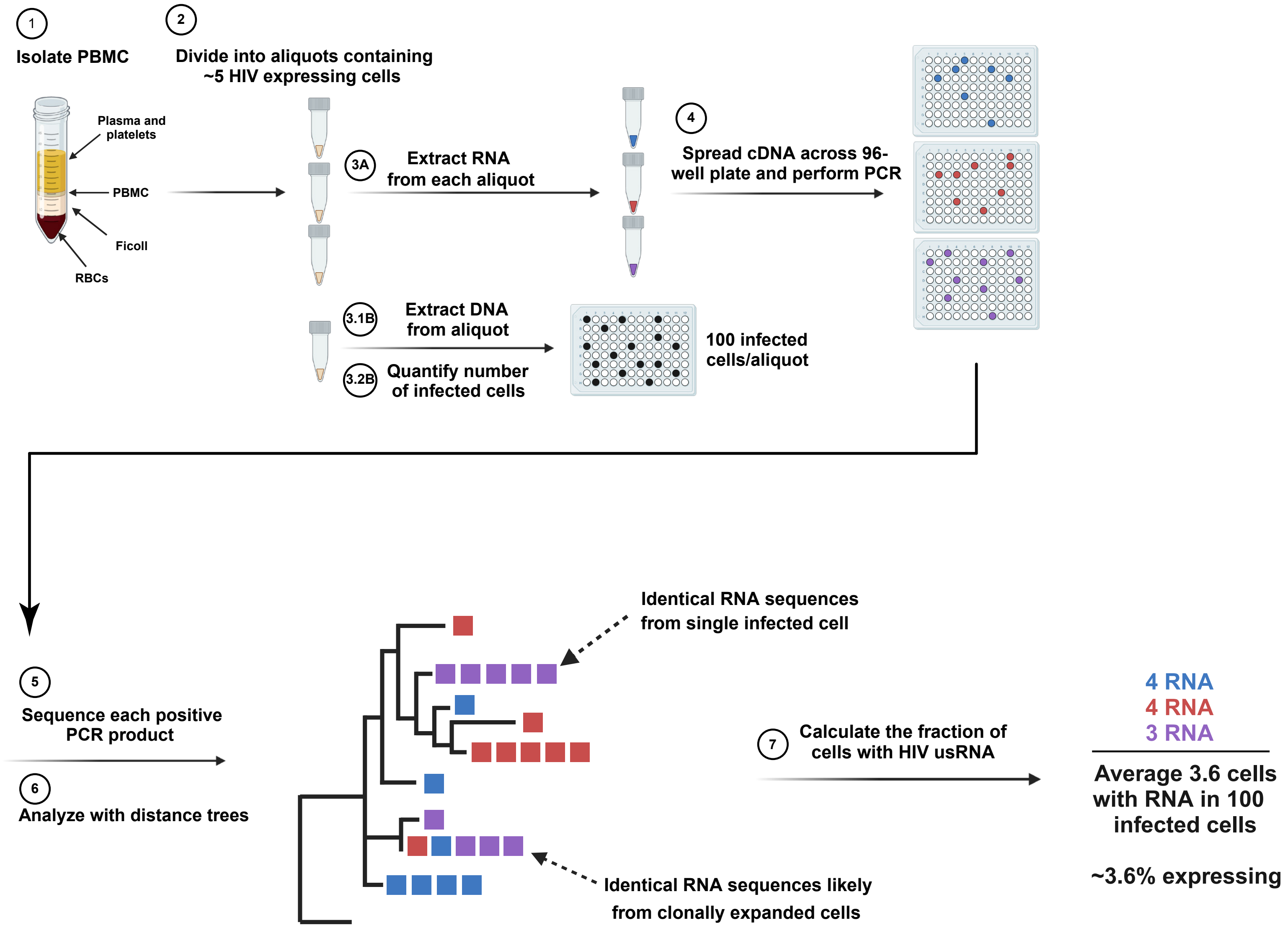

Supplemental Figure 3. Flow chart of CARD-SGS assay. PBMC are isolated from whole blood and separated into separate aliquots. One aliquot is used to determine the number of infected cells per aliquot. Then separate aliquots are made with ~5% HIV usRNA expressing cells per aliquot. RNA is extracted, treated with DNAse, and undergo cDNA with a gene-specific primer. The synthesized cDNA is spread across a 96-well plate and PCR amplified. Following two rounds of PCR targeting the p6-PR-RT region, amplicon detection of positive wells are determined. PCR positive wells are selected and undergo Sanger sequencing. Electropherograms are analyzed through custom in-house scripts, alignment is generated, with neighbor-joining distance trees reconstructed. Analysis of the tress distinguishes identical HIV usRNA sequence from the same aliquot, indicating they derived from a single infected cell; or from different aliquots suggesting they were derived from clonally expanded cells. The sum of the number of unique HIV usRNA populations are divided over the number of infected cells analyzed to determine the fraction of cells expressing HIV usRNA at the time of sampling.
